# Epitopemap: a web application for integrated whole proteome epitope prediction

**DOI:** 10.1186/s12859-015-0659-0

**Published:** 2015-07-14

**Authors:** Damien Farrell, Stephen V Gordon

**Affiliations:** School of Veterinary Medicine, University College Dublin, Belfield, Dublin Ireland

**Keywords:** Epitope, Web interface, Antigen

## Abstract

**Background:**

Predictions of MHC binding affinity are commonly used in immunoinformatics for T cell epitope prediction. There are multiple available methods, some of which provide web access. However there is currently no convenient way to access the results from multiple methods at the same time or to execute predictions for an entire proteome at once.

**Results:**

We designed a web application that allows integration of multiple epitope prediction methods for any number of proteins in a genome. The tool is a front-end for various freely available methods. Features include visualisation of results from multiple predictors within proteins in one plot, genome-wide analysis and estimates of epitope conservation.

**Conclusions:**

We present a self contained web application, Epitopemap, for calculating and viewing epitope predictions with multiple methods. The tool is easy to use and will assist in computational screening of viral or bacterial genomes.

**Electronic supplementary material:**

The online version of this article (doi:10.1186/s12859-015-0659-0) contains supplementary material, which is available to authorized users.

## Background

The antigen presentation pathway is fundamental to the cellular adaptive immune response. During this process certain short peptides processed from self or foreign proteins may be able to activate an immune response. These are called epitopes. Accurate prediction of the peptides that will form antigenic epitopes is essential to rational vaccine design and diagnostics. Prediction of T cell epitopes may be done by taking the protein sequence and estimating the binding affinity of each peptide fragment to MHC class I and II molecules. The strongest binding fragments are then selected. In reality the process is complicated since peptide binding is allele restricted or can be promiscuous.

Given the size of pathogen proteomes and variation between strains it is clear that computational tools are necessary for automated screening and selection of immunological features before experiments are performed. With the availability of whole genomes for many microbial species it is now feasible to computationally search an annotated proteome for likely epitopes, this is the basis of immunoinformatics. Given that the immune system may present sequences from any protein antigen to stimulate T-cell responses, consideration of the entire proteome is necessary for a complete picture of the potential antigen repertoire.

Many MHC binding prediction methods exist for both class I and II and have been comprehensively reviewed [1, 2]. Currently the fastest and most practical are data driven approaches which are trained on existing binding data. All such methods vary in accuracy over MHC loci and alleles, largely depending on the availability of quality data [3]. Binding prediction models for MHC class I and II complexes suffer from the same limitation: a high number of false positives and false negatives. Cut-offs can be raised at the cost of missing potential valuable antigenic peptides. Too low a cut-off yields a larger number of synthetic peptides to test, many of which will be negative. There is therefore a trade-off between discovery and experimental cost. Actual cut-offs chosen will depend on specific requirements of the study. For example, searching a small number of proteins might mean taking the top ranked percentile from each sequence regardless of scores. Given these limitations no method can rely on pure automation. An approach integrating computation and domain knowledge will still be required and end user tools are necessary. One aspect currently lacking are user interfaces that integrate multiple computational methods and allow the factors affecting B cell and T cell immune response to be viewed as a whole.

### Current tools for epitope discovery

A typical requirement for vaccine design is to first examine the immunogenetic diversity of host populations along with an array of different strains of the organism of interest. Predictions over the whole proteome can be made accordingly. The various prediction tools that contribute to this ‘in silico’ stage are available online but must be run individually. Many binding prediction tools have web interfaces but there is no way to conveniently integrate or compare the results from these predictors. Even with tools to automate such a workflow, the user will want to visualise the prediction data in context, such as in relation to other predictions or protein annotations.

Some attempts have been made to provide integrated analysis systems with a user interface. Most are prediction ’pipelines’ and few have extensive user friendly tools. The work described by Bremel et al. [4] is a recent example. This software is based on using a neural network to conduct QSAR regression predictions using various physico-chemical properties of amino acid sequences. It takes into consideration entire proteomes and provides a global standardization procedure for binding scores. However the system is built on the commercial JMP Genomics desktop software and thus not easily amenable to testing. PepVac [5] uses binding prediction to five distinct HLA class I supertypes for promiscuous epitope prediction. It allows upload of genome sequences in fasta format and provides a proteasome cleavage filter. Other commercial desktop software applications for epitope discovery are EpiMatrix [6] and DNASTAR’s Lasergene structural biology suite [7] which includes a tool for linear epitope prediction. Commercial tools may be of high quality but are neither free nor open source, raising issues of reproducibility for academics [8].

We propose a more general but sustainable solution than some of these previous tools. This is to develop a flexible software tool that is not tied down to one particular pipeline or workflow. Such a tool should be built on an open-source license and be available freely from a repository which is independent of the host institution or publisher. Our solution, described here, is a web-based application using web technologies designed for large data applications making the software both scalable and platform independent.

## Implementation

### Web application

**Fig. 1:**
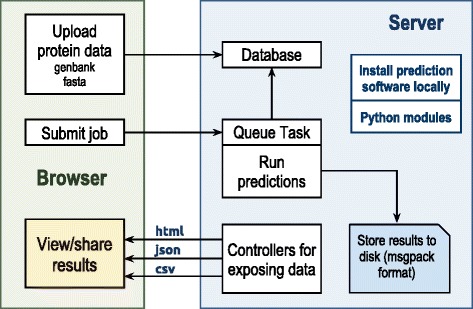
Outline of web application design. A simple schematic of the application shows the typical architecture of the web2py framework. All processing is done on the server side and results presented to the user via html or other formats such as json and csv. Results are not saved directly to the database but persisted to the file system

The web application is implemented using the web2py [9] framework. This software allows rapid prototyping of interactive web applications using a model-view-controller system. web2py is written in Python making it ideal for integrating the previously developed software library described below. The graphical component for sequence viewing is implemented with Bokeh [10], a Python library that provides interactive Javascript plotting. Additional plotting is handled with the mpld3 library [11]. web2py is installed and run on the server and all user interaction takes place in the web browser. This is outlined in Fig. 1.

### Software library

A library for managing epitope predictions was implemented in Python. This provides a standardized programmatic interface for executing several binding prediction methods and processing the results. The results from each method can then be processed and visualized in a consistent manner. To achieve some level of uniformity between prediction methods a base Python class was designed with generic functions. This is then sub-classed to implement the specific functionality of each method, for example, a TepitopePredictor class and so on. Each class must implement a specialised predict method and return data in a standard format. Pandas DataFrames [12] are used for internal data manipulation and for results storage and retrieval to disk. This method allows us to integrate a new prediction method in a relatively straightforward and consistent manner. This mostly consists of wrapping the call to the command line tool and processing the output. This Python library is available as a separate set of modules from its own github repository at https://github.com/dmnfarrell/mhcpredict. It is also provided as part of the web application when downloaded.

## Results and discussion

In the the following we describe the main features provided by the web application.

### Available prediction methods

We have concentrated on using some of the available pan-specific MHC binding prediction methods as summarised here:

- TepitopePan. This is our own Python implementation of TEPITOPEPan [13] and requires no external program to run. This method covers approximately 700 human HLA-DRB (MHC class II) alleles. It is labelled as simply ‘Tepitope’ in the application.
- NetMHCIIpan. netMHCIIpan [14] must be downloaded separately from the website and installed on your system.
- IEDB MHC-I binding prediction tools
- IEDB B-cell epitope prediction tools. A collection of methods to predict linear B cell epitopes based on sequence characteristics of the antigen using amino acid scales and HMMs.

All of these tools are free for academic use. This application was tested for use with netMHCIIpan version 3.0c, IEDB MHC class1 tools version 2.12 and IEDB Antibody Epitope Prediction tool version 1.0. These specific prediction methods were chosen because they are free for academic use, have been widely used and in the case of MHC methods, offer the most allele coverage.

### Adding sequence data

The application is designed to work on sets of proteins, i.e. an entire genome, rather than individual sequences in isolation. Before predictions can be done a file with the annotated proteins must be uploaded. This will be saved to the database. Usually this data will represent the known proteome of a specific species but may be an arbitrary collection of protein sequences.

Annotated protein sequences can currently be added by two methods:

- Genbank format (preferred method). Genbank is used for reasons of consistency and completeness. The locus tag in the genbank format for each protein is usually a unique identifier and short unlike the description field that might be extracted from a fasta file. Genbank files contain features such as gene product or location that are also useful for identifying the protein. Genbank files are available from the NCBI genomes page [15].
- Fasta format. Any collection of sequences contained in a fasta file can be added. They do not have to be related in any way but might represent some subset of a genome or a collection of orthologs. This method relies on correct naming for the each sequence in the description line so is not as reliable as the genbank format. However it is probably more familiar to users. Any sequences added in this way are assigned to the genome ‘other’.

### Submit binding predictions

Predictions are made by submitting via a form that contains all the basic options for the current methods. Multiple methods can be submitted at the same time. Since binding prediction for many proteins can take some time, a job queuing system using the in-built web2py scheduler is employed. Jobs are run in the background without interfering with the normal operation of the application and are queued and run consecutively. A file is stored for each protein so that results can be retrieved on a per protein basis quickly. Once predictions have been saved for a protein they can be immediately viewed, so one does not have wait until the entire job is completed. It is possible to allow jobs to run concurrently to make better use of computers with multiple processors. Thus the application can be scaled to handle a higher throughput of predictions if required. This is detailed in the documentation.

### Thresholds

Selection of predicted binders is usually done based on the percentile rank within a sequence or absolute MHC binding affinity (or whatever score is a proxy for binding). It has recently been shown that absolute binding affinity threshold correlates better with immunogenicity and MHC allele-specific thresholds should be used to improve correlations [16]. In addition Bremel et al. [4] have noted that conclusions about binding affinity cannot be made based on measurements on one protein in isolation. This likely reflects the situation when an infectious organism is digested in an antigen presenting cell and a large repertoire of the resultant peptides are subjected to competitive binding. For this reason we find a good general solution for binder selection is to apply a global percentage threshold on a per-allele basis. This is done by sampling the scores across the proteome for each allele and pre-calculating the quantile value at each percentage level. Thus, rather than taking the top percentage in each protein, the binders shown reflect the results for the other proteins in the genome. This threshold can be altered in the user interface to show more or fewer binders as needed. It should be underlined again that this is a rule of thumb and thresholds must be considered based on the number of sequences being searched, number of epitopes required for an assay and so on.

### Visualization

MHC binding predictors are generally used to make multiple scoring predictions for each allele for an n-mer set of binders. This information is difficult to display in a single plot. One of the primary purposes of this application is to allow the user to quickly visualise the location of predicted epitopes along the protein sequence and compare multiple alleles from several different methods all in one plot. A form allows selection of proteins by locus tag or gene name and a score threshold and minimum allele value. When the form is submitted the right pane is updated with a tabbed pane containing the plot, sequence representation, table of the top binders and display of the gene features from the genbank source.

**Fig. 2:**
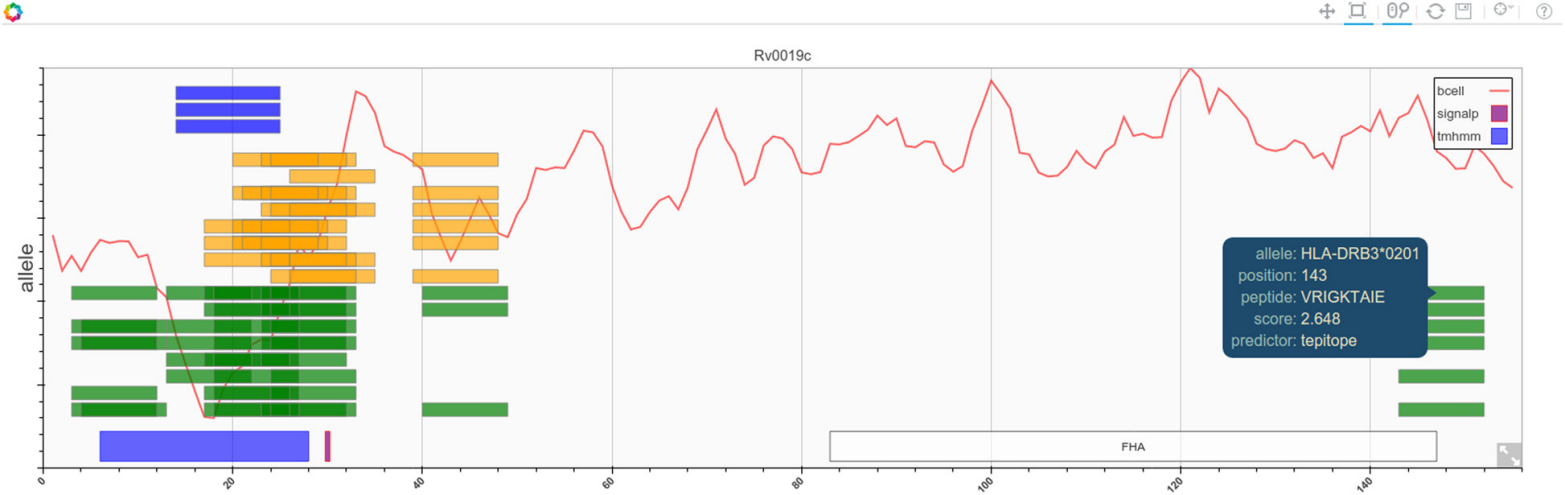
Visualization of results. Plot visualization of multiple binding predictions for a single protein sequence. Tracks for each prediction method are color coded. Protein annotation can be shown if required. A hover tool provides information on each binder

The default visual representation is a set of tracks with bars representing predicted n-mer binders for each allele ordered by position on the sequence as shown in Fig. 2. A useful feature for long sequences is the ability to zoom in and pan left and right. Individual epitope/peptide details can be identified by moving the mouse over the bars. Basic protein annotation can be optionally displayed in the plot. This currently shows predicted transmembrane regions, PFAM annotations and predicted signal peptide cleavage sites along the sequence. This information is not calculated but is retrieved using the SeqDepot [17] REST service. Results can be shared with other users by providing a permanent link to the protein URI in a RESTful manner. For example to share information on protein Rv1886c in genome MTB-H37Rv the following link would be used: http://server/epitopemap/default/protein/myresults/MTB-H37Rv/Rv1886c where ‘myresults’ is the identifier associated with this particular set of predictions.

### Genome-wide analysis

One application of immunoinformatics is to screen out likely candidate antigens from the genome for further study. The approach used here is to perform predictions for all protein sequences and select out potential antigens based on the pattern of predicted binders. The user chooses a set of previously calculated predictions, a global percentage cut-off and minimum number of alleles. All promiscuous binders are calculated along with detection of clusters of these binders along each sequence. Epitope clustering has been previously [18] observed to be an indicator of T cell epitope regions. The result is a table of all proteins with various metrics such as the number of binders per unit length of each protein or the number and highest density of epitope clusters. The table can be sorted by any column. Two additional tables showing the top peptides and clusters are also produced. Users may find it useful to copy these tables into a spreadsheet for further processing. Calculations are done on the fly but results are saved for the chosen settings and can be recomputed quickly.

### Epitope conservation

This module computes the degree of conservation of an epitope within a given protein sequence set at a given identity level. Identity is the degree of correspondence between aligned sequences. The idea is to give a measure of how well conserved a particular epitope is within a subset of similar sequences, for example representing a set of pathogenic strains. Our method uses the NCBI online Blast [19] service to retrieve orthologous matches. By default all sequences are retrieved with an expect value lower than 10 and can then be filtered by percentage identity. Sequences are checked for duplication. It may be preferable or necessary to refine blast queries to narrow down the sequence set to a specific taxon or species. This can be done using an Entrez query option which restricts the search to a subset of entries from the NCBI nr (non-redundant) protein database fitting the requirement of the Entrez query. The result is a table of aligned sequences and a list of percentage conservation of each promiscuous epitope at the chosen identity threshold. Also produced is a plot of minimum identity versus conservation for each epitope. The concept is to provide a ranking of epitopes shared with similar species or strains. A comparable tool is available online as part of the Immune Epitope Database and Analysis Resources (IEDB) [20]. That tool requires the user to provide sequences manually and provides a more detailed amino-acid breakdown of conservation.

### Integration of experimental data

Experimental data characterizing antibody and T cell epitopes studied in humans and other animal species is important for benchmarking predictions and cross checking during vaccine development. The main source for this data is the IEDB [21] but there are also smaller databases. We have added a basic search interface for accessing these datasets using the pepdata library [22]. This is a Python interface to commonly used immunology and bioinformatics datasets (i.e. IEDB, cancer antigens, TCGA mutant peptides). The tool currently consists of a search form with tabulated results. Further development of thisfeature is discussed below.

### Deployment

We envisage this application typically being used as a local application in a single-user context. However installation of the software dependencies may be a challenge for some users. For those wishing to install their own copy of the application it may be easier to use a virtual appliance. This is a virtual machine OS with all software pre-installed. We can make such a virtual image available on request. More details are given in the documentation. Advanced users can consult the deployment recipes in the web2py book [23].

### Discussion

Rather than a prediction pipeline, this is an end-user visual tool that will assist with the inherent ’needle in a haystack’ problem of epitope based vaccine design. The application will be useful in the crucial first filtering step to narrow down experimental choices. MHC binding prediction is only one step in the search for suitable epitopes. In practice multiple other factors limit the peptides which actually serve as epitopes. Proteolytic cleavage is a critical process which determines which peptides are available to be bound by MHC molecules. The T-cell receptor binding process is another determinant of which epitope will activate an immune response. Some of these other predictors of antigenicity have yet to be integrated into this application but will appear in a future version. There are also a number of other epitope filtering strategies in the literature [24, 25] that could be included to exploit the data structures we have designed to build the framework. The detection of epitope clusters is just one example.

The integration of experimental data is a non-trivial task since it requires correct identification of the source protein sequence (antigen) that the epitope in question belongs to so that predictions can be compared. The IEDB meta data is usually sufficient for this but other databases may not be. Our web interface currently allows the public databases to be searched but is still in development. It is planned that the user will be able to do a search and then immediately submit the source protein sequences for prediction. Then the predictions will be overlaid with the experimental epitopes. This opens the possibility of using the tool for quick benchmarking.

This software is a flexible platform designed to be extensible by anyone familiar with Python programming. We have focused on providing a tool that can be installed locally and/or accessed from the server over a network. We encourage those wishing to use the program to install it themselves. However a public server is available at http://enzyme.ucd.ie/epitopemap to allow review and testing. Users are welcome to request a guest login and try the application there. Detailed support information is available as a web page inside the application. An additional movie file is also provided that outlines the operation of the software by example (see Additional file 1).

## Conclusion

In this study, we introduce Epitopemap, a web based application for integrated execution, visualisation and analysis of MHC binding predictions in a flexible and user friendly way. The software has broad applicability to any pathogenic proteome. The tool is novel in providing a unifying framework for currently existing prediction methods. There are many ways to exploit the genome-scale integrative approach, the genome analysis tool included is one example. The ability to visualise coincident features of multiple predictors within proteins in one plot will provide rapid insight into immunological features and should save significant time. Users can tailor their studies to specific allele groups for MHC-I and MHC-II predictors allowing viewing of results for specific populations. As updates to the standard predictors are released these can be integrated into the application with little or no change. We intend to add more features, such as experimental data integration, in the near future.

## Availability and requirements

- **Project name:** Epitopemap
- **Project home page:** dmnfarrell.github.io/epitopemap
- **Operating system(s):** Platform independent
- **Programming language:** Python
- **Other requirements:** web2py, ncbi-blast+ tools, python-pandas, python-bokeh
- **License:** Apache License v2.0
- **Any restrictions to use by non-academics:** non-academic license needed to use external binding prediction methods such as netMHCIIpan

## Additional file

Additional file 1
**Screencast showing the basic usage of the Epitopemap web application.**

## Abbreviations

MHC: Major histocompatibility complex
APC: Antigen presenting cell
IEDB: Immune epitope database and analysis resources
BLAST: Basic local alignment search tool
HMM: Hidden Markov model
QSAR: Quantitative structure activity relationship
REST: Representational state transfer
